# Cerebral control of winking before and after learning: An event‐related fMRI study

**DOI:** 10.1002/brb3.1483

**Published:** 2019-11-21

**Authors:** Chou‐Ching K. Lin, Kuo‐Jung Lee, Chih‐Hsu Huang, Yung‐Nien Sun

**Affiliations:** ^1^ Department of Neurology National Cheng Kung University Hospital College of Medicine National Cheng Kung University Tainan Taiwan; ^2^ Department of Statistics and Institute of Data Science National Cheng Kung University Tainan Taiwan; ^3^ Department of Computer Science & Information Engineering National Cheng Kung University Tainan Taiwan

**Keywords:** blinking, functional MRI, learning, winking

## Abstract

**Introduction:**

The main purpose of this study was to investigate the cerebral areas responsible for winking by observing the activation pattern and learning effects on cerebral cortices by comparing differences in activation pattern during winking before and after learning.

**Methods:**

Sixty‐three subjects were recruited, including 22 (11 males; 11 females) who could wink bilaterally and 41 (14 males; 27 females) who could wink unilaterally. Event‐related functional magnetic resonance was performed. The subjects were asked to blink and wink according to projected instructions as the events for image analysis. The activation pattern was obtained by contrasting with the baseline images without eyelid movements. Those who could only wink unilaterally were asked to train themselves to wink the other eye. For those who succeeded (*n* = 24), another imaging study was performed and the results were compared with those before training.

**Results and conclusion:**

Left winking resulted in activation in the left frontal lobe, while right winking resulted in activation in bilateral frontal lobes with predominance on the right side. For the subjects capable of only winking unilaterally, learning to wink on the other side activated similar cortical areas to those in the subjects capable of bilateral winking without training.

## Graphical abstract

Winking one eye can be learned easily, so that winking may be a good model to investigate the learning effects of controlling facial muscles on the cerebral cortex. Left winking showed activation in the left frontal lobe, while right winking showed activation in the bilateral frontal lobes with predominance on the right side. For the subjects capable of only winking unilaterally, learning to wink on the other side activated similar cortical areas to those in the subjects capable of bilateral winking without training. Learning to wink is achieved through strengthening the activation of inherent cortical areas, not by recruiting and changing the function of other cortical areas.

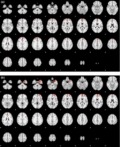

## 1 INTRODUCTION

Eyelid movements include bilateral blinking and unilateral winking. While blinking is a routine and functionally indispensable movement for eyes, winking is a selective movement for specific purposes, such as aiming during shooting and flirtation. The central neural control of voluntary eyelid movements has not been well established due to limited evidence. Several studies have reported that impairment of volitional eyelid movements can be caused by localized brain lesions, such as in frontal cortical areas or the corticospinal system (Almallouhi & Dale, 2018; Esteban, Traba, & Prieto, 2004; Thon, Grossmann, & Bhattacharyya, 2017). In addition, Mazzone et al. (2010) found that frontostriatal activity was increased in persons with Tourette syndrome during the inhibition of eye blinks compared with normal subjects. This finding implicitly suggests that the frontal lobe is involved in the control of blinking. In a study combining electrooculography and fMRI, Chung et al. (Chung, Yoon, Song, & Park, 2006) showed that bilateral parahippocampal, precentral gyrus, and left supplementary motor areas were activated during voluntary blinking, whereas medial/superior frontal, precentral, cingulate, precuneus, and superior temporal gyrus were activated during voluntary blink inhibition in 12 healthy right‐handed subjects (five women and seven men) aged 22 ± 1.5 years. In addition, Hanakawa et al. (Hanakawa, Dimyan, & Hallett, 2008) found that volitional blink tasks activated the medial frontal areas, bilateral opercular areas extending into the precentral gyrus on the right, and the right temporoparietal areas in an fMRI study of 10 healthy right‐handed subjects (six women and four men) aged 23–36 years.

Even fewer studies have investigated the control of winking. van Koningsbruggen et al. (van Koningsbruggen, Peelen, Davies, & Rafal, 2012) reported that different neural mechanisms affected the ability to wink one eye in an fMRI study of a group of bilateral stroke patients compared with healthy subjects. They also found that the frontal eye field (FEF) played a critical role in voluntary unilateral eye closure. Korn et al. (Korn, Reith, & Becker, 2004) reported a 78‐year‐old man who had had an ischemic stroke which damaged the right rostral part of the callosal forceps and was unable to voluntarily close his left eyelid. The neurological conditions responsible for unilateral wink apraxia are still poorly understood. People who suffer from the disability find it difficult or impossible to perform certain eye winks, even though they can blink and accept training to move their eyelids.

The main goal of this study was to unravel the cortical area activated by the nondeliberately and deliberately learned winking. This study may have both basic and clinical implications. Basically, the results answer the question whether deliberate learning activates inherent or other cortical areas. Clinically, for those locked‐in patients, learning of bilateral winking may facilitate and enrich the communication with the outside world. We conducted an event‐related fMRI study in a group of young subjects, some of whom were initially unable to wink with one eye. Those who were unable to wink with either their left or right eye were instructed to train themselves and eventually succeeded in gaining the ability to wink bilaterally. After training, they underwent an fMRI study again in order to investigate changes in the activation pattern of the brain by comparing the patterns before and after training.

## 2 MATERIALS AND METHODS

### 2.1 Participants

Sixty‐eight subjects aged from 20 to 40 years (mean = 24.6 years; standard deviation = 3.55) were recruited. The inclusion criteria included grossly normal mentality, no neurological or psychiatric disorders or history of seizures, not taking any medications that might affect nervous system function, ability to cooperate and the ability to wink at least one eye. This study complied with the Declaration of Helsinki, and the study protocol was approved by the Ethics Committee of National Cheng Kung University Hospital. Before entering the study, written informed consent was obtained from all participants.

### 2.2 Experimental design

In the experiment, the participant saw a sequence of stimuli projected onto a screen by a computer. These stimuli were blinking/winking tasks consisting of three conditions: voluntary bilateral blinking, right winking, and left winking. The participant was instructed to respond as quickly and accurately as possible and voluntarily blink/wink without maintaining the blink/wink for too long. Each action for a stimulus usually lasted for <1 s. These three conditions were presented randomly and consecutive conditions were separated semirandomly by a fixation period of 7 ± 2 s, in order to avoid expectation and rhythmicity. The subjects were told to open their eyes as long as possible but to blink spontaneously during the fixation periods where a cross was presented in the middle of the screen and which served as baseline in the statistical analysis. Each study session consisted of four runs, with each run lasting 6 min. To ensure that the participants understood the test procedures, they first practiced in a mock fMRI scanner before being tested in the actual scanner.

The subjects who could only wink one eye were asked to practice winking the other eye by themselves at home. Once they had learned to wink the other eye, they underwent another fMRI study following the same protocol. In general, the subjects learned to wink the other eye within 1 month.

### 2.3 Image acquisition

MR imaging was performed on a Siemens 3T Trio scanner using a 32‐channel head coil. Functional images were acquired with a gradient‐echo echo‐planar imaging sequence in 30 contiguous axial slices covering the whole brain with TR = 2,000 ms, TE = 30 ms, 64 × 64 pixels in a slice, 4‐mm slice thickness, and 4 × 4 mm^2^ in‐plane resolution. To reach equilibrium of magnetization and allow the signal to return to the baseline after the last stimulus, five dummy (10 s of fixation) scan volumes were added at the beginning and six dummy scan volumes (12 s of fixation) were added at the end of a run. Each BOLD series consisted of 125 image volumes and 122 were used in the image analysis. In addition to the functional images, structural T1‐weighted images were collected using a magnetization‐prepared rapid acquisition gradient echo sequence, with TR = 2,350 ms, TE = 3.4 ms, 256 × 256 pixels in a slice, field of view = 25.6 cm, and 1 mm slice thickness, resulting in 1 mm^3^ isotropic resolution. The image data can be given to any researcher upon reasonable request to the corresponding author.

### 2.4 Image processing

The raw fMRI data required preprocessing before statistical analysis to remove extraneous sources of variability that may have occurred during acquisition. The preprocessing steps included motion correction using the MCFLIRT subroutine (Jenkinson, Bannister, Brady, & Smith, 2002), slice timing correction using Fourier‐space time‐series phase‐shifting, nonbrain signal removal using BET (Smith, 2002), denoising using the MELODIC subroutine, Gaussian spatial smoothing using a Gaussian kernel with a full width at half maximum of 5 mm (Mikl et al., 2008), grand‐mean intensity normalization of the entire 4D dataset using a single multiplicative factor, and high‐pass filter with a cutoff equal to 60 s to remove low‐frequency drifts (Friston et al., 2000). Registration to the high‐resolution structural and/or standard space images was carried out using the FLIRT subroutine (Jenkinson et al., 2002; Jenkinson & Smith, 2001). Registration from the high‐resolution structural images to the standard space was then further refined using the FNIRT subroutine nonlinear registration. All of these fMRI data processing procedures and the following statistical analyses were carried out using the freeware FEAT (FMRI Expert Analysis Tool), version 6.00, which is part of FSL (FMRIB Software Library, http://www.fmrib.ox.ac.uk/fsl).

### 2.5 Experimental design and statistical analyses

The data were fit to a general linear model (Friston, 2004) and examined. The following three actions, blinking and left and right winking, were separately modeled by convolution of two gamma functions in the general linear model. Motion parameters, age, and gender were included in the analysis as covariates of no interest. We used two‐stage analysis, where summary estimates of model parameters were obtained at the subject level by fitting the voxel‐wise linear model and then used in the second stage model at the group/population level. In other words, runs were pooled on a per subject basis using a fixed‐effects model. A mixed‐effects group analysis was then conducted using FMRIB FLAME stages 1 and 2, in which relevant lower‐level contrasts were combined. In stage 2, the two‐sample *t* test was used. For each contrast, we reported cortical regions as activated with a height threshold of *Z* > 3.1 and a cluster probability of *p* < .05, thereby correcting the whole‐brain multiple comparisons based on Gaussian random field theory (Worsley et al., 2002). The code for image analyses can be given to any interested researcher upon reasonable request to the corresponding author.

To investigate the cerebral control of eye blinking and winking, we compared the brain activation images of voluntary bilateral blinking, left winking, and right winking to those of the fixation baseline. In addition, we compared the participants who could wink bilaterally to those who could only wink with one eye.

## 3 RESULTS

After the initial examination of the quality of the fMRI data, five subjects were found to have made excessive head motions and were thus removed from later analyses. The remaining 63 subjects were entered into the analysis. The characteristics and distribution of the subjects are shown in Table 1. One female subject who could wink bilaterally was left‐handed, and all of the other subjects were right‐handed. About half of the subjects learned how to wink bilaterally within 1 month, which showed that bilateral winking could be learned easily. Laterality of winking may not be a genetically determined trait, as bilateral, left unilateral, and right unilateral winking were roughly equally present in this group of mostly right‐handed subjects.

**Table 1:** Characteristics of the study subjects

Wink ability	Bilateral	Unilateral				Total
		Left		Right		
		Learned	Not‐learned	Learned	Not‐learned	
Male	11	4	3	4	3	25
Female	11	10	4	6	7	38
Total	22	14	7	10	10	63

Note

“Learned” denotes that the subjects who learned to wink the eye after training. “Not‐learned” denotes that the subjects were still unable to wink the eye after training.

### 3.1 Blinking versus baseline

**Figure 1:**
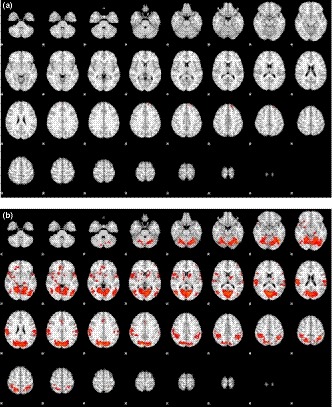
Map of (a) activation (blinking > baseline) and (b) deactivation areas (blinking < baseline) in blinking

To study the brain areas related to eye blinking, the functional images of blinking tasks were contrasted with those of the baseline without training in all 63 subjects (25 males and 38 females). The activation areas (blinking > baseline) included primarily the bilateral frontal lobes with an emphasis on the left side (Figure 1a). In contrast, deactivation (blinking < baseline) was noted in many regions including parietal, occipital, and temporal lobes (Figure 1b). Of note, the deactivation areas were much more widespread than the activation areas. Table 2 lists all the activation/deactivation areas and the corresponding statistics.

**Table 2:** Activation areas of voluntary blinks contrasted to baseline in all subjects

Task	Cerebral area	Brodmann area	Talairach labels (*x*, *y*, *z*) (mm)	Maximum *z*‐value
Blink > Baseline	Frontal lobe		(−12, 50, 42)	4.33
	Superior frontal gyrus		(−18, 48, 38)	4.25
Blink < Baseline	Superior parietal lobe	7A (L & R)	(−20, −70, 34)	4.51
	L/R cerebellum		(14, −54, −10)	4.15
	Inferior parietal lobe		(60, −38, 32)	4.10
	L/R occipital lobe		(−50, −78, 2)	4.46
	Temporal lobe		(−42, −64, −16)	4.58
	Parietal lobe		(−40, −52, 42)	4.38

Abbreviations: L, left; R, right.

### 3.2 Winking versus baseline

**Figure 2:**
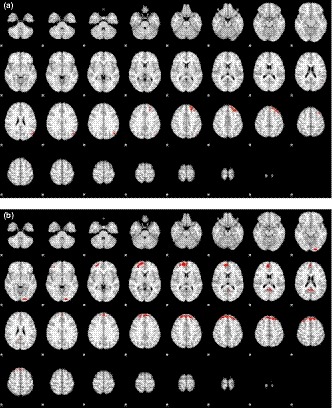
Map of activation area (winking > baseline) in (a) left and (b) right winking of all participating subjects by contrasting to their own baseline

In contrasting, the successful left winking to the baseline (*n* = 43 subjects; male/female: 18/25), the activated brain areas were the left frontal and parietal lobes (Figure 2a). On the other hand, by contrasting the successful right winking to the baseline (*n* = 42 subjects; (male/female: 18/24), the activated brain areas were bilateral frontal and occipital lobes (Figure 2b), with an emphasis on the right frontal area. Table 3 lists all the activation areas and the corresponding statistics.

**Table 3:** Activation areas of performing winks contrasted to baseline in all subjects

Task	Cerebral area	Brodmann area	Talairach labels (*x*, *y*, *z*) (mm)	Maximum *z*‐value
L wink > Baseline	Middle/Superior frontal lobe	8L	(−28, 34, 46)	6.39
	Parietal lobe		(−52, −70, 26)	5.24
R wink < Baseline	Frontal lobe		(34, 44, −4)	6.76
	Parietal lobe/posterior cingulate		(−4, −54, 14)	4.36
	Occipital	18L	(−24, −94, −4)	5.44

Abbreviations: L, left; R, right.

We also contrasted the winking tasks to the blinking tasks, including left winking > blinking, left winking < blinking, right winking > blinking, and right winking < blinking. When the height threshold was *Z* > 3.1 and the cluster probability was *p* < .05, no areas were activated in any of the combinations.

### 3.3 Successful versus failed winking

**Figure 3:**
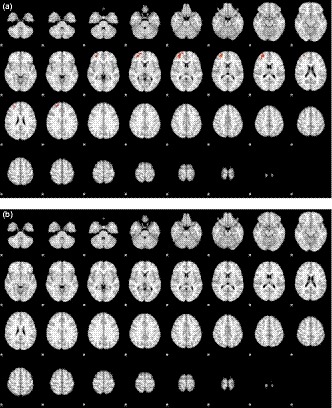
Map of activation area in (a) left and (b) right winking by comparing those capable and incapable of winking

We contrasted the subjects who could not wink to those who could wink to investigate what additional brain regions were activated. The results showed that the frontal lobe and middle frontal gyrus were more active during the left winking task in the subjects who could not wink with the left eye than in those who could (Figure 3a). However, when comparing the subjects who could not wink with the right eye to those who could, no further activation areas were detected (Figure 3b). Table 4 lists all the activation areas and the corresponding statistics.

**Table 4:** Comparison of activation areas of performing winks contrasted to baseline in those capable and incapable of the requested wink without training

Capable > incapable	Cerebral area	Brodmann area	Talairach labels (*x*, *y*, *z*) (mm)	Maximum *z*‐value
L wink	L frontal gyrus	10L	(34, 50, 8)	5.80
R wink	—		—	—

Abbreviations: L, left; R, right.

### 3.4 Effects of learning

**Figure 4:**
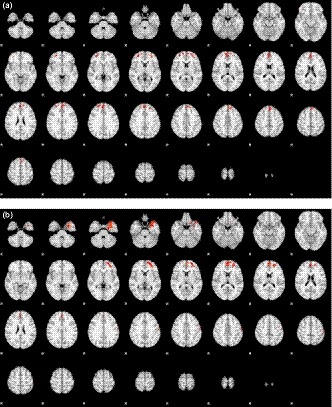
Map of activation area (winking > baseline) in left winking for those incapable of left winking (a) before and (b) after having learned the action

**Figure 5:**
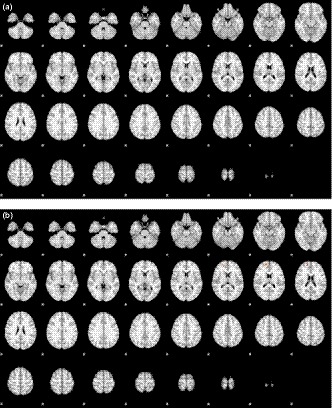
Map of activation area (winking > baseline) in right winking for those incapable of right winking (a) before and (b) after having learned the action

In this part, we focused on the subjects who were initially unable to wink in one eye but learned to do so after training. Before learning to wink with the left eye, the activated areas included bilateral frontal and midline frontal areas (Figure 4a), whereas after learning, the activated areas only included the midline and left frontal areas (Figure 4b). The activated areas after learning to wink with the left eye were similar to those in the subjects who could wink with the left eye without training, except that the midline frontal area was also activated in the trained group. With regards to right winking, while there were no activated areas before learning (Figure 5a), a small spot in the left frontal area was activated after learning (Figure 5b). The activated areas after learning to wink with the right eye were much smaller than those in the subjects who could wink with the right eye without training, although activation in both groups was focused on the right frontal lobe. Table 5 lists all the activation areas and the corresponding statistics.

**Table 5:** Comparison of activation areas of performing winks before and after learning the requested wink

Task		Cerebral area	Brodmann area	Talairach labels (*x*, *y*, *z*) (mm)	Maximum *z*‐value
L wink	Before	L frontal lobe		(−18, 44, 40)	6.55
		Middle frontal lobe		(−32, 50, 4)	5.79
		R frontal lobe		(32, 50, −8)	6.39
	After	L frontal lobe		(−32, 44, 2)	8.11
		L temporal lobe	38L	(−36, 18, −30)	7.60
		L postcentral gyrus	4L	(−62, −12, 36)	6.54
R wink	Before	—		—	—
	After	Frontal lobe/superior frontal gyrus		(16, 52, 16)	5.24

Abbreviations: L, left; R, right.

## 4 DISCUSSION

The main results of this study are as follows. Voluntary blinking was associated with small areas of activation in bilateral frontal lobes with predominance on the left side, left winking was associated with activation in the left frontal lobe, and right winking was associated with activation in the bilateral frontal lobes with predominance on the right side. For those who could not wink with the left eye, trying to do so was associated with activation in the bilateral frontal lobes, and, after having learned, successful left winking was associated with activation in the left frontal lobes. For those who could not wink with the right eye, trying to do so was not associated with any activation at all, and, after having learned, successful right winking was associated with activation in the right frontal lobe.

Part of bilateral visual and cerebellar area were deactivated in blinking. Because the baseline was watching the screen with eyes opening, it is expected that blinking would cause deactivation in the visual area. A study (Friston, 2004), supporting this expectation, showed that blinking momentarily activated default mode network and the visual areas. However, deactivation of cerebellum is unexpected. Most of the past studies about the role of cerebellum in blinking concentrated on the blink reflex and showed activation of cerebellum. However, the neural mechanism of blink reflex is very different from that of the voluntary blink investigated in this study. In addition, many studies of voluntary blinking by functional MRI did not or only partially imaged the cerebellum (Nakano, Kato, Morito, Itoi, & Kitazawa,^2013^). We think a possible explanation for the deactivation of bilateral cerebellum is the distinct connection of cerebellum with intrinsic connectivity networks. Our results revealed that the deactivation mainly localized to the vermis and deep nuclei, which were shown in a study (van Eimeren et al., 2001) to be related to the default mode network and the sensorimotor network.

Only a small activation area in frontal lobes was noted in the blinking tasks, which is different from the results of previous studies (Chung et al., 2006; Habas et al., 2009; Hanakawa et al., 2008), that showed activation of supplementary and primary motor areas. One possible explanation for this discrepancy may be the difference in the experimental paradigms. We instructed our subjects to perform modest blinking for a short time, which may not have been sufficient to provoke activation strong enough to be detected. In the study by Kato and Miyauchi (Kato & Miyauchi, 2003), the subjects were asked to blink every 0.5–2 s in a self‐paced style. Supplementary motor area was reported to be activated in voluntary rhythmic or semirhythmic movements, expected or planned movements (Grahn & Brett, 2007; Kato & Miyauchi, 2003). We deliberately used jittered interval between consecutive movements so that the cue could not be expected. This might be one reason that SMA was not activated in our study. In the study of Hanakawa et al. (Hanakawa et al., 2008), the blinking was a willful, voluntary movement that involved contraction of the orbicularis oculi muscles but not of other body parts. It is also possible that the control of modest eye closure, which is mainly achieved by the relaxation of the levator palpebrae superioris muscle, is different from that of forceful eye closure, which is achieved by contracting the orbicularis oculi muscle. The activation area for subtle movements such as blinking may also be less consistent with regards to spatial distribution among subjects, so that group averaging statistically canceled out the significance of activation areas.

The activation patterns of right and left winking (Figure 2) were different in this study, in that the activation areas of left winking were localized to the left hemisphere, while those of right winking were bilateral and more widespread. This implies that left winking requires less effort, which seems to be consistent with general findings that humans are more apt to use left winking when aiming and signaling. This conclusion may only be applicable to right‐handed subjects, because all but one of the subjects in the present study was right‐handed.

The tactics adopted by the subjects when they were asked to wink an eye that they could not seemed to be different for the two sides. For left winking, in addition to the left frontal area, the right frontal area was also activated, that is, the activation became bilateral. For right winking, no additional area was activated, indicating the effort or activation area was similar. This implies that when the action was less familiar or less skillful, more cortical areas in bilateral hemispheres were recruited to achieve the goal. After having learned the skill, the activated area reduced to one hemisphere (Figure 4b). The activated areas for learned left winking were similar to those in the subjects capable of left winking without training. On the other hand, the activated areas for learned right winking were much smaller than those in the subjects capable of right winking without training (Figure 5b). The combined results of left and right winking indicated that learning to wink was achieved through strengthening the connection between the voluntary control center and the inherent cortical area for eyelid movements, and not by recruiting and changing the function of other cortical areas.

Both right and left winking resulted in more activation than blinking. However, whether the activated areas were excitatory or inhibitory for eye closure is unclear. For example, activation of left frontal areas during left winking may have been associated with left eye closure or the inhibition of right eye closure. As the pure blinking task did not result in other activated areas as in other studies, we cannot verify which mechanism is applicable in this study.

## 5 CONCLUSION

Left winking resulted in activation in the left frontal lobe, while right winking resulted in activation in the bilateral frontal lobes with predominance on the right side. For the subjects capable of only winking unilaterally, learning to wink on the other side activated similar cortical areas to those in the subjects capable of bilateral winking without training. The results indicated that learning to wink is achieved by strengthening the connection between the voluntary control center and the inherent cortical area for eyelid movements.

## CONFLICT OF INTEREST

The authors declare no competing financial interests.

## AUTHOR CONTRIBUTIONS

CCL recruited subjects and wrote the first draft of this manuscript, CHH designed MRI protocol and performed MRI study, KJL and YNS performed image and statistical analyses, and all authors reviewed and approved the manuscript.

## Data Availability

The data that support the findings of this study are available on reasonable request from the corresponding author. The data are not publicly available due to the data size and privacy.
